# Human PAPS Synthase Isoforms Are Dynamically Regulated Enzymes with Access to Nucleus and Cytoplasm

**DOI:** 10.1371/journal.pone.0029559

**Published:** 2012-01-05

**Authors:** Elisabeth Schröder, Lena Gebel, Andrey A. Eremeev, Jessica Morgner, Daniel Grum, Shirley K. Knauer, Peter Bayer, Jonathan W. Mueller

**Affiliations:** 1 Department of Structural and Medicinal Biochemistry, Faculty of Biology, Centre for Medical Biotechnology, University of Duisburg-Essen, Essen, Germany; 2 Department of Molecular Biology II, Faculty of Biology, Centre for Medical Biotechnology, University of Duisburg-Essen, Essen, Germany; National Institute for Medical Research-Medical Research Council London, United Kingdom

## Abstract

In higher eukaryotes, PAPS synthases are the only enzymes producing the essential sulphate-donor 3′-phospho-adenosine-5′-phosphosulphate (PAPS). Recently, PAPS synthases have been associated with several genetic diseases and retroviral infection. To improve our understanding of their pathobiological functions, we analysed the intracellular localisation of the two human PAPS synthases, PAPSS1 and PAPSS2. For both enzymes, we observed pronounced heterogeneity in their subcellular localisation. PAPSS1 was predominantly nuclear, whereas PAPSS2 localised mainly within the cytoplasm. Treatment with the nuclear export inhibitor leptomycin B had little effect on their localisation. However, a mutagenesis screen revealed an Arg-Arg motif at the kinase interface exhibiting export activity. Notably, both isoforms contain a conserved N-terminal basic Lys-Lys-Xaa-Lys motif indispensable for their nuclear localisation. This nuclear localisation signal was more efficient in PAPSS1 than in PAPSS2. The activities of the identified localisation signals were confirmed by microinjection studies. Collectively, we describe unusual localisation signals of both PAPS synthase isoforms, mobile enzymes capable of executing their function in the cytoplasm as well as in the nucleus.

## Introduction

Central to all biological sulphation reactions in eukaryotes is the conversion of the very stable oxy-anion sulphate to the high-energy sulphate donor 3′-phospho-adenosine-5′-phosphosulphate (PAPS). Sulphation of a variety of biomolecules can be understood as a high-affinity, low-capacity conjugation system where the availability of the precursor PAPS is rate-limiting [1]. A vast number of sulphotransferases has been described with 53 current entries for human enzymes in Entrez Gene by now [2]. Part of this enzymatic apparatus is required to create the highly complex extracellular sulphated carbohydrates [3]. Other sulphotransferases are crucial for phase II detoxification of xenobiotics during biotransformation [4].

The many and diverse sulphotransferases are localised to the Golgi apparatus [5], the cytoplasm [6] and even the nucleus [7]. They are fed by only two PAPS producing enzymes in vertebrates – the bifunctional PAPS synthases 1 and 2 (PAPSS1/2) [8], [9] consisting of an N-terminal adenosine-5′-phosphosulphate (APS) kinase domain and a C-terminal ATP sulphurylase domain connected by a short irregular linker [10]. Lower animals seem to have only one PAPS synthase gene, and this single enzyme is essential at least for the worm *Caenorhabditis elegans* [11]. An involvement of human and mouse PAPS synthase 2 in bone and cartilage malformation is firmly established [9], [12]. More recently, PAPS synthases have also been implicated in steroid metabolism [13] and hepatitis B infection [14].

PAPSS1 has only very recently been reported to play a yet undefined role in retroviral infection [15]. Using retroviral insertion mutagenesis in CHO-K1 cells, a mutant cell line was isolated with about 10fold reduction in infectivity by murine leukaemia virus (MLV). This cell line had a mutation in the PAPS synthase 1 gene and showed a defect in an intracellular step of retroviral replication. Clearly, reduced HIV infection rates were not due to reduced sulphation of the HIV co-receptor CCR5 [16]. Within the nucleus, the requirement for PAPS was mapped to a step during viral DNA integration into the host genome that has a subsequent effect upon the level of expression of viral genes. The exact nature of this PAPS-dependent process is yet unknown, but it also affects gene transcription from the long terminal repeat regions of human immunodeficiency virus 1 (HIV-1) [15].

All the different phenotypes caused by PAPS synthase deficiencies were reported in the context of a wild-type gene for the other PAPS synthase isoform. A major question at this point is why the two isoforms cannot complement for each other. Both, Bruce et al. [15] and Noordam et al. [13] referred at this point to the different cellular localisation of the two proteins. There is only one previous study on the localisation of PAPS synthases [17] reporting human PAPSS1 as a nuclear protein. Besides, murine PAPSS2 was also studied and was found to reside exclusively in the cytoplasm in the absence of overexpressed PAPSS1. Upon co-expression of PAPSS1, however, localisation of mouse PAPSS2 was shifted towards the nucleus [17]. Recent evidence revealed that this process may occur via the formation of high-affinity PAPS synthase heterodimers [18].

We show that sub-cellular localisation of the human PAPS synthases is more intricate than reported previously. Although both PAPSS synthase proteins are able to shuttle between nucleus and cytosol, we could confirm a general trend of more nuclear PAPSS1 and more cytosolic PAPSS2 protein in our study. Both proteins were only marginally affected in their localisation after prolonged treatment with the export inhibitor leptomycin B pointing to an exportin1-independent export mechanism. By mutagenesis, a positively charged α-helical segment within the APS kinase domain of both proteins was identified that also showed export activity in microinjection experiments. Finally, we identified an N-terminal KKxK motif as a strong, active nuclear localisation signal conserved in bi-functional PAPS synthases.

## Results and Discussion

### PAPS synthases show pronounced heterogeneity in their sub-cellular localisation

**Figure 1:**
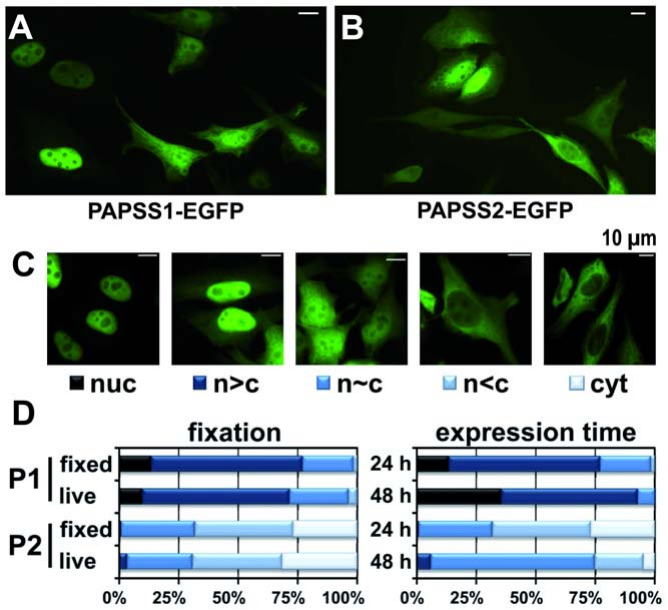
Localisation of PAPS synthase wild-type fusion proteins. **A and B**, microscopic images of PAPSS1- and PAPSS2-EGFP fusions. Transfected HeLa cells were fixed upon 24 h of fluorescent protein expression. Within every sample, cells with varying protein localisation could be observed. **C**, schematic for classifying localisation of PAPS synthase fusion proteins and their mutants. **D**, localisation pattern for PAPS synthase wild-type proteins with respect to the way of fixation and the expression time. A total of 1026 cells for PAPSS1-EGFP (P1) and 1942 cells for PAPSS2B-EGFP (P2) were evaluated, respectively, in fixed HeLa cells after 24 hours of expression. For all other conditions at least 200 cells were scored except PAPSS2-EGFP in living HeLa cells (n = 122). EGFP, enhanced green fluorescent protein.

Previously, it was reported that PAPS synthases differ in their sub-cellular localisation [17]. However, when expressing fusions of fluorescent proteins and PAPS synthases in HeLa cells, we observed remarkable heterogeneity of localisation for both enzymes (**Figure 1A and B**). Hence, we classified a large number of cells into five categories according to the localisation of their protein fluorescence. The five classes used in this study are depicted in **Figure 1C**.

### PAPSS1 is predominantly nuclear and PAPSS2 cytosolic

**Figure 2:**
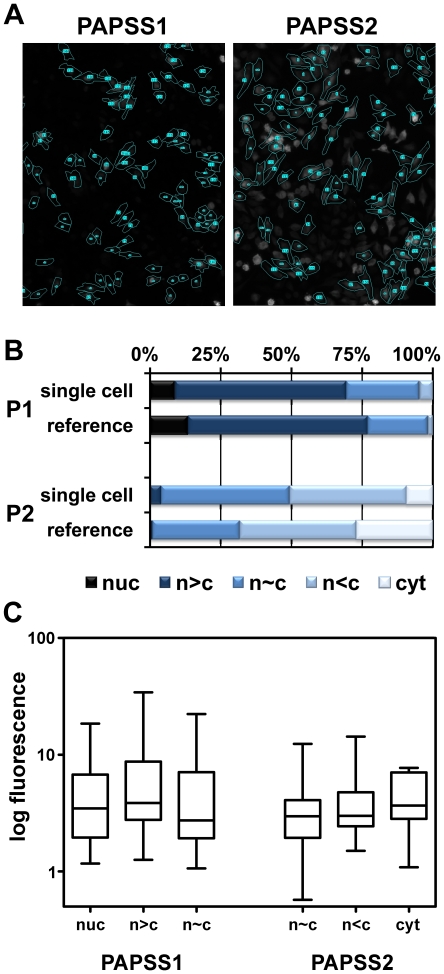
Heterogeneity of localisation does not correlate with PAPS synthase-EGFP expression levels. **A**, microscopic images of fixed HeLa cells transfected with PAPSS1- and PAPSS2-EGFP fusion plasmids were analysed on a single-cell level using ImageJ 1.45. Therefore, cells were scored according to the schematic of Figure 1 and cell borders were defined as regions of interest (ROI). Then, cell surface as well as mean fluorescence intensity was measured separately for each cell followed by background correction. **B**, using single cell analysis, 147 cells were measured by for PAPSS1-EGFP and 150 for PAPSS2-EGFP. The classification of these ensembles recapitulates the heterogeneous localisation pattern for both PAPS synthases reported in Figure 1. **C**, multiplication of cell area and mean fluorescence intensity results in a dimension-less fluorescence. This value represents the total fluorescence of each cell and is assumed to correlate with the expression level of EGFP fusion proteins. For the three major classes of each ensemble, fluorescence was plotted in a box-and-whisker plot. No correlation between expression level/fluorescence and cellular localisation could be derived.

To test our scoring system and the robustness of the method, several microscopic samples were prepared consecutively varying different parameters. The general trend in all our experiments was that PAPSS1-EGFP was predominantly located to the nucleus, and more of the PAPSS2-EGFP fluorescence could be detected in the cytosol. Evaluation of transiently transfected living and fixed cells gave comparable results (**Figure 1D**). Hence, the following investigations were performed on fixed microscopic samples. Prolonged expression of the respective EGFP fusions (48 h vs. 24 h) resulted in a shift towards a more nuclear localisation for both PAPSS1 and -S2 (**Figure 1D**). PAPSS2-EGFP was additionally expressed for 72 hours, not resulting in any significant further nuclear accumulation (data not shown). At this point, the effect of the fusion protein was also tested. DsRed_mono_ fusion proteins of PAPSS1 and PAPSS2 showed comparable expression levels to the EGFP proteins, but were shifted more towards the cytoplasm (**Information S1**). Most of the subsequent experiments were done using the EGFP vector background. To test for cell line-specific effects, PAPS synthase fusion proteins were additionally studied in HEK 293 and CHO cells. In these cell lines, localisation did not differ largely from the pattern observed in HeLa cells (**Information S1**). Under all conditions tested, both PAPS synthases were found to a certain extent in the nucleus and the cytoplasm. To test whether the heterogeneous cellular localisation described here was an indirect effect of varying expression levels of the EGFP fusion proteins, the total fluorescence signal was determined as the product of one cell's area and its average fluorescent intensity for ensembles of PAPSS1- and PAPSS2-EGFP expressing HeLa cells. No correlation was observed between expression level and observed localisation pattern (**Figure 2**). The molecular weights of 70 kDa for monomeric and, even more, 140 kDa for dimeric PAPS synthase proteins preclude passive diffusion because of the size exclusion limit of the nuclear pore complex at around 40 kDa. Hence, the localisation pattern described here may indicate the involvement of active nuclear transport processes.

### Inhibition of CRM1-dependent nuclear export has little effect on cellular localisation of both PAPS synthases

**Figure 3:**
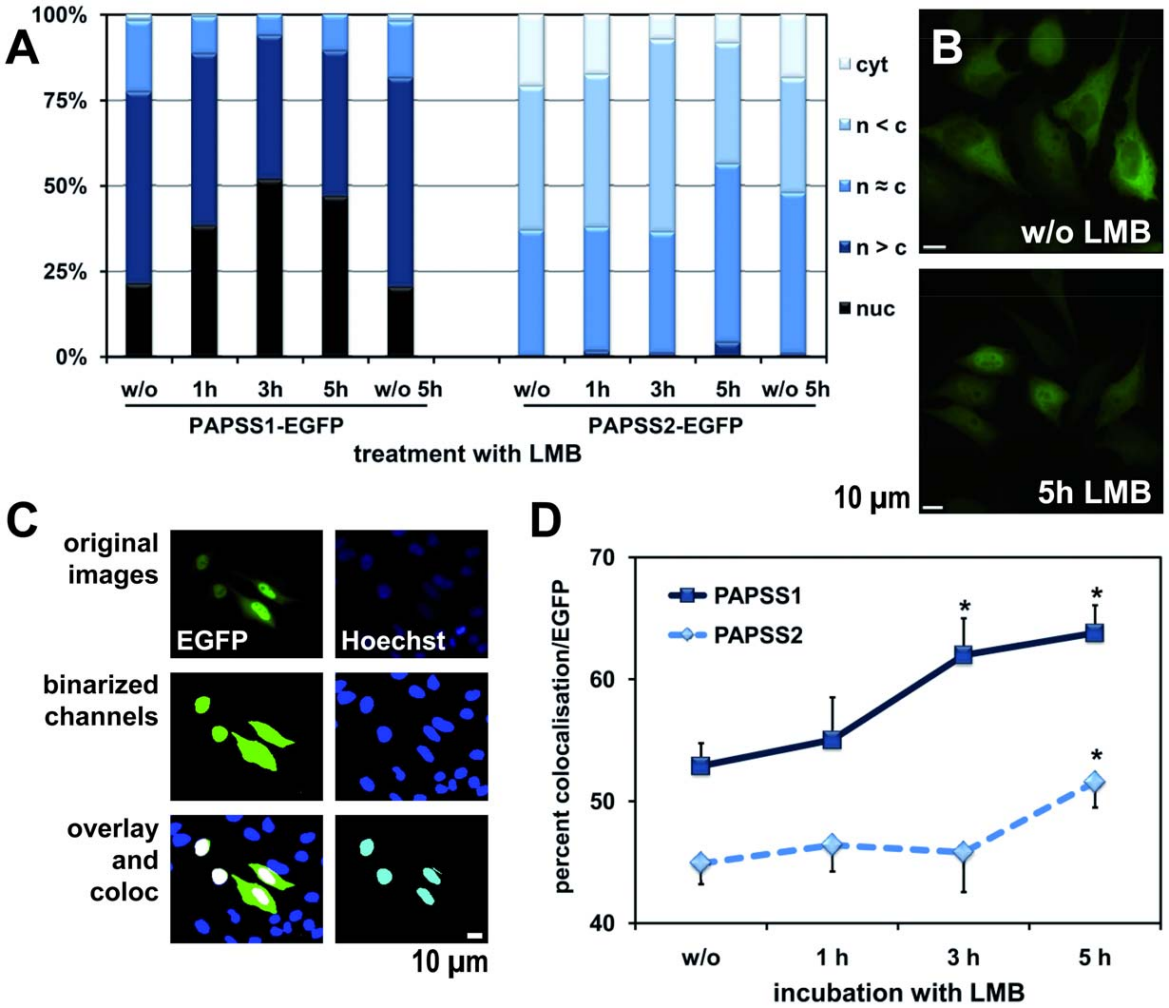
Effect of LMB treatment on PAPS synthase localisation. **A**, localisation patterns for PAPS synthase wild-type fusion proteins following LMB treatment. HeLa cells expressing PAPSS1- and PAPSS2-EGFP fusion proteins were treated with the export inhibitor leptomycin B (LMB) at a concentration of 10 nM for prolonged periods of time. At least 200 cells were visually evaluated per lane according to the scheme from figure 1. **B**, examples of PAPSS1-EGFP expressing HeLa cells after 5 h LMB or mock treatment demonstrating increased nuclear accumulation caused by LMB. **C**, alternative assessment of quantitative localisation after binarisation. Original images are shown before and after binarisation. In this instance, the extracted parameters were: area of Hoechst fluorescence (16%), EGFP fluorescence (8.5%) and co-localisation (3.8%), co-localisation relative to Hoechst (23%) and EGFP (44%) areas (Coloc_rel_H and Coloc_rel_E, respectively) as well as the ratio of EGFP and Hoechst areas (0.53) reflecting transformation efficiency. The area of Coloc_rel_E was found to be the most robust parameter for nuclear localisation of our PAPS synthase-EGFP fusion proteins. **D**, effect of LMB treatment on PAPS synthase-EGFP fusion protein localisation measured according to **C**. The area of Coloc_rel_E significantly increases for both PAPSS1- and PAPSS2-EGFP proteins upon prolonged LMB treatment. *p values for unpaired two-tailed t-test relative to no LMB treatment: PAPSS1-EGFP with 3 h LMB (0.034) and 5 h LMB (0.006) as well as for PAPSS2B-EGFP with 5 h LMB treatment (0.038).

The majority of nuclear export is mediated by the nuclear export factor exportin1/CRM1. This export receptor can be specifically inhibited by the natural product leptomycin B (LMB). Hence, we determined whether LMB changed the localisation patterns for the PAPS synthase proteins. Nuclear localisation was found to be moderately enhanced after prolonged LMB incubation for both PAPS synthase proteins. Even though the effects were relatively weak, and the shifts towards nuclear localisation rather incomplete, the accumulation could not be detected in untreated cells (**Figure 3A and B**). We additionally determined the percentage of co-localisation of the green fluorescence of the PAPS synthase-EGFP fusions with Hoechst nuclear staining after binarisation of the microscopic images as an unbiased computation-based approached (**Figure 3C**). Indeed, the area of co-localised fluorescence relative to the EGFP signal increased significantly upon LMB treatment for both PAPS synthase proteins.

Nevertheless, a leucine-rich nuclear export signal (NES) following the recently proposed consensus for Rev-class NESs [19] was found within the sequence of PAPSS2 (**Information S1**). The crystal structure 1X6V of full length PAPSS1 proves the corresponding sequence motif to be surface exposed and in mainly alpha-helical conformation (**Information S1**). In microinjection experiments, we observed that fluorescent GST-GFP fusion proteins containing the proposed NESs were slowly exported into the cytoplasm (**Information S1**). The remarkably strong HIV-1 Rev NES was also assayed for comparison (see below). In agreement with existing data [20], this motif caused significantly stronger export activity than the leucine-rich motifs described here. The addition of some hydrophobic sequence to the GST-GFP transport substrate might have caused a direct interaction with the FG repeats of the nuclear pores and thereby accelerated nuclear pore passage slightly. Therefore, CRM1-dependent nuclear export seems not to play a major role in determining the cellular localisation of PAPS synthases.

### A mutagenesis screen to identify potential NLS and NES motifs

**Figure 4:**
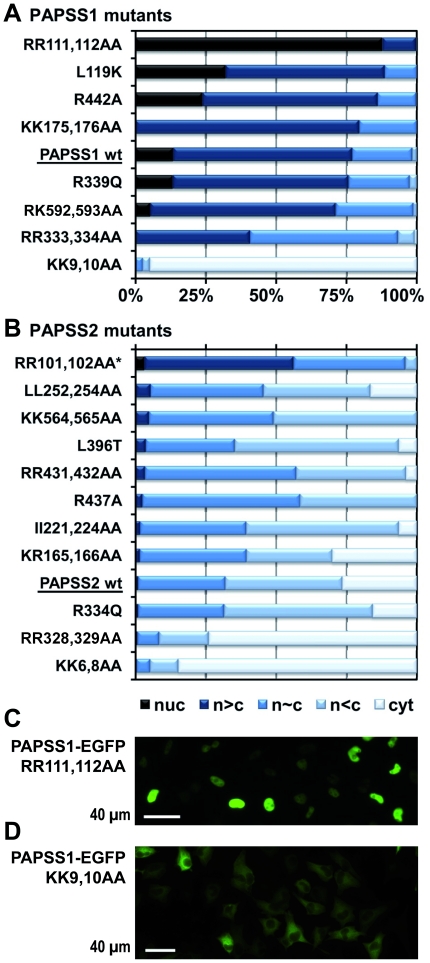
Mutation screen for localisation signals in PAPS synthases. **A and B**, phenotype of all mutants evaluated in this study for PAPSS1 and -S2. Potential NLS and NES signals were targeted by alanine mutagenesis. This also included the putative NESs around Ile221/Ile224 and Leu252/Leu254 in PAPSS2. At least 200 cells were scored for each mutant. All mutants are arranged according to the sum of the two fractions “nuc” and “n>c”. *This mutant was evaluated in a DsRed_mono_ context due to very weak expression of the respective EGFP fusion protein. **C and D**, microscopic images of PAPSS1-EGFP RR111,112AA as well as PAPSS1 KK9,10AA showing the strong and nearly uniform phenotype of these two mutants.

Next, we mutated putative localisation signals in PAPS synthase 1 and 2 followed by quantitative assessment of their patterns of cellular localisation. As PAPS synthases do not reside exclusively in the cytoplasm, and no classical nuclear localisation signals (NLS) could be found, we targeted (I) patches of at least two arginine and/or lysine residues as parts of putative NLS motifs that are (II) clearly surface-exposed in the available crystal structure 1X6V of PAPSS1 [10]. These criteria resulted in a total of 13 putative NLS mutants of PAPS synthase-EGFP fusions. Moreover, we mutated some residues of human PAPS synthase 2 to their murine counterparts as a previous study has reported an exclusively cytoplasmic murine PAPSS2 protein [17]. Leu252 and Leu254, mentioned above, as well as Ile221 and Ile224 were also changed to alanines to test their effect as putative NES motifs within the full length proteins. All mutants were generated by site-directed mutagenesis in the background of our PAPS synthase-EGFP expression constructs, expressed in HeLa cells and scored for their cellular localisation patterns (**Figures 4A and B**).

Remarkably, the most severe shifts in cellular localisation were induced by the RR111,112AA and KK9,10AA mutations in PAPSS1 (**Figure 4C and D**) as the following: RR111,112AA resulted in a nearly complete nuclear accumulation of the PAPSS1-EGFP fusion protein. The KK9,10AA led to the opposite effect. That is why the respective mutants were further studied and described in more detail below. The corresponding mutations in PAPSS2 (RR101,102AA and KK6,8AA) showed similarly shifted localisation patterns. Changing amino acids to their murine counterparts (L396T and R334Q in PAPSS2; R339Q in PAPSS1) had no effect on cellular localisation patterns of PAPS synthases. Weakly decreased nuclear localisation was observed for the putative NLS mutants RR333,334AA in PAPSS1 and RR328,329AA in PAPSS2. Other mutants aimed at targeting NLS motifs resulted in slightly increased nuclear localisation (KK175,176AA and R442A in PAPSS1 as well as R437A in PAPSS2). As the mutant II221,224AA in PAPSS2 also localised like the wild type, these residues seemed not to belong to a functional export signal. Mutating leucine residues L252 and L254 to alanines resulted in slightly increased nuclear accumulation of PAPSS2. For the mutant PAPSS2-EGFP fusion proteins that showed the largest shifts in cellular localisation, we determined expression levels by western blotting (**Information S1**). As these were found to be nearly identical at similar rates of transfection, we concluded that their strongly shifted cellular localisation patterns are direct effects of the mutations and not indirect effects of protein expression levels. In sum, our mutagenesis screen revealed a novel sequence motif mediating export activity containing R111 and R112 in PAPSS1 as well as the counteracting comparably strong NLS in the very N-terminus containing K9 and K10 in PAPSS1.

### Transport activity in a heterologous background: an α-helical motif with export activity

The strong effect of mutants RR111,112AA in PAPSS1 and RR101,102AA in PAPSS2, respectively, was a surprising finding from our mutagenesis screen. These mutations were thought to obliterate a putative NLS and, hence, expected to result in increased cytoplasmic localisation of the respective EGFP fusions. Though, the opposite was observed: In nearly all cells, PAPSS1-EGFP RR111,112AA was exclusively found within the nucleus (**Figure 4C**). In the dimeric crystal structure 1X6V of PAPSS1 [10], these residues are found within a long α-helix at the interface of the two APS kinase domains. As both arginines and alanines are known α-helix-promoting residues, NPS predictions [21] did not point to markedly changed secondary structure for the double-alanine mutant.

Nevertheless, we hypothesised that our mutations may compromise formation of PAPS synthase dimers that are known to have nano-molar affinity [18]. Subsequent exposure of unwanted residues might interfere with proper cellular sorting. To test this possibility, PAPSS1 wild-type protein and the respective mutant RR111,112AA were expressed in a bacterial host and analysed for their dimerisation behaviour by analytical gel filtration. L119K, a second mutation within this long helix that enhanced nuclear localisation of PAPSS1-EGFP, was tested in the same way. All these PAPSS1 proteins clearly behaved like dimers in gel filtration and their localisation was confirmed following binarisation (**Table 1**). Of note, all proteins were loaded onto the column at concentrations of 2-10 µM and are at most three-fold diluted during the gel filtration run, indicating the K_D_s of all these mutants still to be far below 1 µM.

**Table 1:** Apparent molecular weight of PAPSS1 mutants.

PAPS synthase tested	apparent Mw [kDa]	co-localisation [%/EGFP]
PAPSS1 wild type	133	45.2±6.4
L119K	137	64.9±3.0
RR111,112AA	133	73.2±4.5

All proteins were loaded at a concentration of 2–10 µM on the gel filtration column. Dilution during chromatography is three-fold at most. Co-localisation was assessed after binarisation of images as described in the methods section.

**Figure 5:**
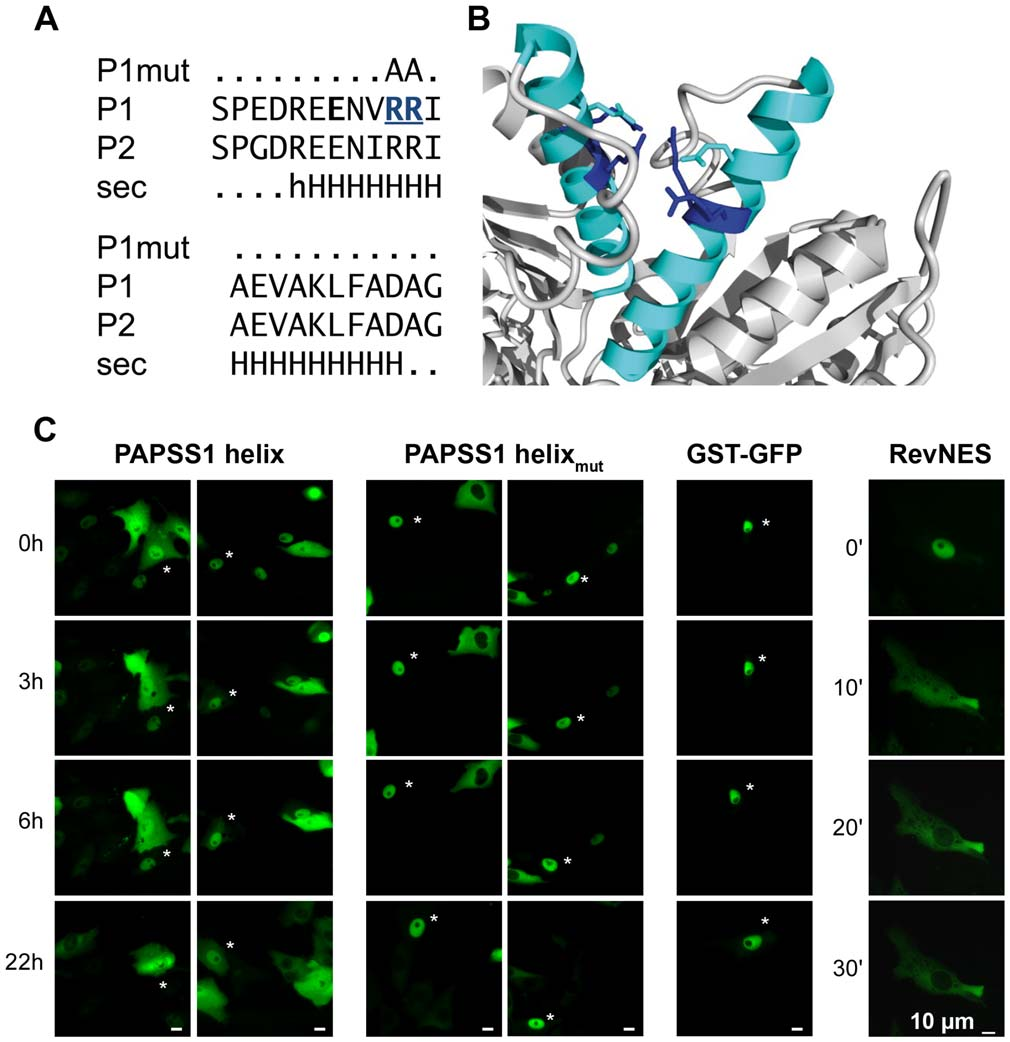
A positively charged helical structure in PAPSS1 shows export activity. **A**, alignment of residues Ser102-Gly124 of PAPSS1 (P1) and the respective Ser92-Gly114 sequence of PAPSS2 (P2). The two arginine residues targeted by alanine mutation are highlighted. NPS secondary structure consensus prediction confirms an extended α-helical conformation; “.”, coil; h/H, weak/prominent helical propensity. **B**, mapping of this motif (cyan) on the interface of the APS kinase dimer within the PAPSS1 crystal structure 1X6V. The two arginine residues are shown in stick representation (blue). Additionally, Glu108 is shown in stick representation (cyan). This residue may be necessary for charge compensation. **C**, a recombinant GST-PAPSS1-Helix-GFP protein showed export activity upon microinjection into the nuclei of Vero cells at the indicated time points (left panels). In contrast, mutation of two arginine residues completely abolished nuclear export (middle panel), and the respective substrate remained nuclear as microinjected GST-GFP alone under the same experimental conditions (right panel). As a positive control, the Rev NES of HIV (LQLPPLERLTL) was completely exported from the nucleus within 30 min following microinjection. Approximately 50 cells were injected, and representative examples are shown.

Next, we studied the α-helical motif independent from full length PAPS synthase proteins in a heterologous background of recombinant GST-GFP proteins by microinjection experiments [22]. The potential export signal was expressed as fusions with glutathione S-transferase and GFP (GST-NES-GFP), purified and injected into the nuclei of Vero cells, and transport was monitored over time by live-cell fluorescence microscopy. There, the surface-exposed helix surrounding Arg111 and Arg112 of PAPSS1 (**Figure 5A and B**) also showed NES activity independent from the proteinous context of PAPS synthases, as the corresponding helix-containing transport substrate was slowly, but quantitatively exported into the cytoplasm (**Figure 5C**). As the tested 23 aa-comprising sequence motif was highly conserved between the two human PAPS synthases (91% sequence identity), these experiments were solely performed with the peptide from PAPSS1. As a control, GST-GFP was inactive under identical experimental conditions, assuring that our system is not flawed by passive diffusion (**Figure 5C**). The highly potent Rev NES was also injected as positive control. Notably, the observed export activity was completely abolished by replacing the same two arginine residues by alanines (Helix_mut_, **Figure 5A and C**) that were also mutated in our screen. This mutation made the α-helical transport signal as inactive as the GST-GFP control under identical experimental conditions.

The surrounding amino acid sequence of this helix could not be matched to any leucine-rich NES type described so far, neither PKI- nor Rev-type, and overall cellular localisation was only marginally affected by treatment with the CRM1 inhibitor leptomycin B. Several CRM1-independent NES sequences have been reported [23]–[26], but there is no clear consensus amongst them. Likewise, export of the human aci-reductone dioxygenase (hADI1) via a non-canonical NES composed of multiple basic amino-acid residues was not mediated via CRM-1 [27]. There, substitution of the basic residues with alanines abolished NES activity in the protein context of hADI1 similar to our observations with PAPSS1. Basic residues contributing to nuclear export might be indicative for an involvement of exportin 7 in nuclear export [28], though the exact nuclear export signal for this export receptor has not been definitively established [29].

### The N-terminus of both PAPS synthases contains a highly conserved KKxK motif

**Figure 6:**
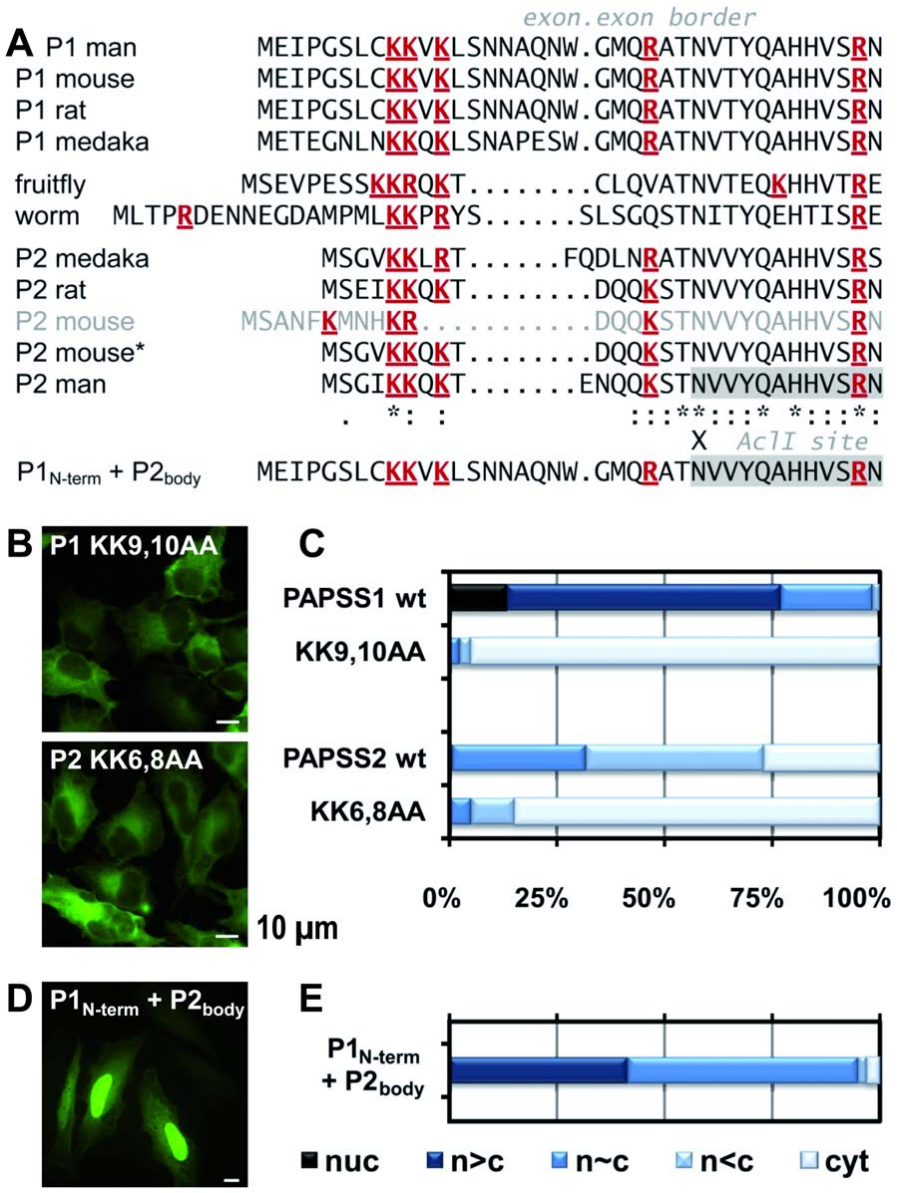
A conserved NLS within the N-terminus of PAPS synthases. **A**, alignment of the N-terminal protein sequence of PAPS synthases from several organisms. PAPS synthase N-termini were aligned with regard to their conserved N-terminal KKxK motif and the beginning of the conserved sequence of the globular APS kinase domain. Although this motif should be regarded as K(K/R)x(K/R) only due to the sequences from fruitfly and worm, we adhere to KKxK in the text for simplicity. All positively charged amino acids are written in red and underlined. An exon-exon border is indicated for all sequences derived from Ensembl. For mouse PAPS synthase 2 an alternative N-terminal sequence, P2 mouse*, is given that is supported by 14 different EST sequences. The sequence for the swapping construct of the PAPSS1 N-terminal sequence and the remaining part (body) of PAPSS2 is given below. Sequences used: human PAPSS1 (P1) [RefSeq: NM_005443], mouse P1 [RefSeq: NM_011863], rat P1 [RefSeq: NM_001106471], medaka P1 [Ensembl: ENSORLP00000008587], splice form RA from *Drosophila melanogaster* [FlyBase: FBgn0020389], the PPS-1 protein from *C. elegans* [RefSeq: NM_069456/Wormbase: T14G10.1], medaka P2 [Ensembl: ENSORLG00000006251], rat P2 [RefSeq: NM_001106375], mouse P2 [RefSeq: NM_011864**.3**], mouse P2* (**Information S1**) and human PAPSS2B [RefSeq: NM_001015880]. **B**, HeLa cells expressing PAPS synthase point mutants within the conserved KKxK motif as EGFP fusions. All samples were fixed after 24 h expression. **C**, quantitative evaluation of mutants shown in B and comparison to the wild-type proteins PAPSS1-EGFP and PAPSS2-EGFP. **D and E**, a swapping construct with the N-terminal sequence of PAPSS1 and the PAPSS2-EGFP body expressed in HeLa cells shows clear nuclear accumulation relative to the PAPSS2-EGFP wild type.

Our screen revealed strong phenotypes for mutating an N-terminal KKxK motif both in PAPSS1 and -S2 that may constitute a nuclear localisation signal (NLS). In agreement with this finding, it has been shown previously that deletion of the N-terminal 21 amino acids of PAPS synthase 1 abrogates nuclear localisation [17]. To look at this motif in more detail, the N-termini of the human proteins were aligned with the respective sequences of other PAPS synthases (**Figure 6A**). Among these was PPS-1, the unique PAPS synthase from the roundworm *Caenorhabditis elegans* that carries a KKxR motif and has been described as nuclear protein [11].

On the contrary, an ectopically expressed mouse PAPSS2-EGFP fusion was detected primarily in the cytosol [17]. In that study, the coding sequence of AF052453 was used, which is identical to the current RefSeq entry NM_011864**.3**. The encoded protein (P2 mouse written in gray in **Figure 6A**) contains some positively charged residues in the form of a KxxxKR motif, but not the above mentioned KKxK motif. To compare this sequence to more distantly related sequences, also the PAPS synthase sequences from rat, medaka (*Oryzias latipes*) and the fruitfly *Drosophila melanogaster*, all containing the KKxK motif, were added. Is the mouse PAPSS2 sequence then an eminent exception? To test this, we used the second exon of murine PAPSS2 to search the mouse expressed sequence tag (EST) collection at NCBI using BLASTn. A total of 15 EST sequences from various murine tissues were retrieved that are listed in **Table 2** together with their encoded N-terminal protein sequence. Only one of these ESTs (CA496923) supports RefSeq entry NM_011864**.3**. The other 14 EST are in favour of an N-terminus that is highly homologous to the rat and human proteins (marked with an asterisk in **Figure 6A**). Interestingly, the end of the variable N-terminal stretch and the beginning of the conserved part of PAPS synthases correlate with a conserved exon-exon border (**Figure 6A**) derived from Ensembl. In agreement with this, both mRNA sequences may result from regular splicing events: the sequence Besset and colleagues [17] have used originates from an exon 13.7 kB upstream of exon2. The new EST-supported variant would require the removal by splicing of a 38 kB intron between this sequence and exon2. Hence, the murine PAPS synthase 2 variant investigated previously [17] may resemble our PAPSS2 KK6,8AA mutant explaining the formerly described strong cytoplasmic phenotype. Assuming the 15 retrieved ESTs in **Table 2** represent a quantitative picture of cellular transcript levels (*in silico* northern blot), the resulting cytoplasmic protein is a minor isoform, and the splice variant encoding the conserved N-terminus represents the more abundant mRNA species. Future research may show whether an additional layer of regulated splicing contributes *in vivo* to the intricate PAPS synthase localisation patterns described here. For now, we conclude that a KKxK motif is remarkably conserved in all aligned PAPS synthase N-terminal protein sequences embedded within a variable sequence context.

**Table 2:** Supporting evidence for an alternative mouse PAPSS2 N-terminus.

Acc. No.	mouse tissue (mRNA source)	size [bp]	encoded N-terminal protein sequence
EST sequences			
BB599382.1	adult pancreas islet cells	262	MSGVKKQK**K** DQQKSTNVVYQAHHVSRNK
BF788130.1	kidney	935	MSGVKKQKT DQQKSTNVVYQAHHVSRNK
BP766402.1	pancreatic islet (C57BL/6)	514	MSGVKKQKT DQQKSTNVVYQAHHVSRNK
BY038761.1	pooled tissues	372	MSGVKKQKT DQQKSTNVVYQAHHVSRNK
BY039169.1	pooled tissues	473	MSGVKKQKT DQQKSTNVVYQAHHVSRNK
BY039481.1	pooled tissues	357	MSGVKKQKT DQQKSTNVVYQAHHVSRNK
BY048063.1	pooled tissues	371	MSGVKKQKT DQQKSTNVVYQAHHVSRNK
BY050605.1	pooled tissues	383	MSGVKKQKT DQQKSTNVVYQAHHVSRNK
BY057385.1	pooled tissues	345	MSGVKKQKT DQQKSTNVVYQAHHVSRNK
BY224664.1	spleen lymphocyte	429	MSGVKKQKT DQQKSTNVVYQAHHVSRNK
BY225041.1	spleen lymphocyte	392	M**X**GVKKQKT DQQKSTNVVYQAHHVSRNK
BY323396.1	synovial fibroblasts	336	MSGVKKQKT DQQKSTNVVYQAHHVSRNK
BY331608.1	synovial fibroblasts	333	MSGVKKQKT DQQKSTNVVYQAHHVSRNK
BY722941.1	adult female vagina	642	MSGVKKQKT DQQKSTNVVYQAHHVSRNK
CA496923.1	kidney	834	**MSANFKMNHKR** DQQKSTNVVYQAHHVSRNK
cDNAs			
AK137675	adult female vagina	2048	MSGVKKQKT DQQKSTNVVYQAHHVSRNK
AF052456.1	[9]	1996	**MSANSKMNHKR** DQQKSTNVVYQAHHVSRNK
BC090997.1	[41]	3652	**MSANFKMNHKR** DQQKSTNVVYQAHHVSRNK

The mouse EST database at GenBank was searched using BLASTN using the following exon2 sequence of murine PAPS synthase: *gaccagcaaa aatccaccaa tgtggtctac caggcccatc atgtgagcag gaacaagaga ggacaagtgg ttggaaccag gggaggattc cgaggatgta ccgtgtggct aaca* .

All 15 hits are listed in this table. Moreover, the available cDNAs are listed. EST, expressed sequence tag.

Both human PAPS synthase isoforms were nearly completely excluded from the nucleus (**Figure 6B**), when the above described KKxK motif was mutated. Notably, nuclear exclusion seemed to be slightly more pronounced for the PAPSS1-EGFP protein (**Figure 6C**) indicating that the N-terminus of PAPSS1 may be somewhat more effective in directing PAPS synthases to the nucleus. Hence, we transferred this N-terminal segment of PAPS synthase 1 to the body of the protein of PAPSS2-EGFP (the resulting sequence P1_N-term_+P2_body_ is also given in **Figure 6A**). Clearly, nuclear localisation was enhanced for this protein chimera relative to the parental PAPSS2-EGFP protein, but still less than for PAPSS1-EGFP (**Figure 6D and E**).

**Figure 7:**
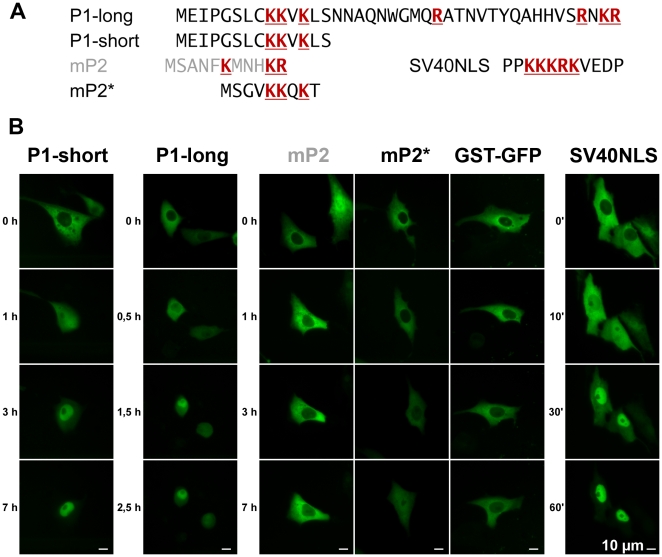
The KKxK motif shows import activity. Two peptide variants of the N-terminus of human PAPSS1 differing in length (long and short) as well as the peptides encoded by the first exon of murine PAPSS2 were tested as GST-GFP transport substrates in microinjection experiments. **A**, peptide sequences of the transport substrates tested. **B**, both recombinant GST-PAPSS1_N-term_-GFP proteins showed import activity upon microinjection into the nuclei of Vero cells at the indicated time points (left panels). In contrast, an N-terminal peptide derived from murine PAPSS2 (mP2) remained cytosolic as microinjected GST-GFP alone under the same experimental conditions, whereas an N-terminal peptide of an alternatively spliced murine PAPSS2 (mP2*) displayed weak import activity (middle panel). As a positive control, the nuclear localisation signal of the SV40 T-antigen (SV40NLS) was completely imported into the nucleus within 60 min following microinjection (right panel). Approximately 50 cells were injected, and representative examples are shown.

To validate the activity of the proposed N-terminal nuclear import sequence, we again applied microinjection of the KKxK motif within the GST-GFP transport substrate system. There, we analysed a short and an extended N-terminal peptide from human PAPSS1 (**Figure 7**). Clearly, both constructs were imported into the nuclei of Vero cells. The NLS of the T antigen of SV40 was used as positive control and was imported even faster. An N-terminal peptide derived from the coding sequence AF052453 of murine PAPSS2 remained within the cytosol like the negative control GST-GFP. However, an N-terminal peptide of an alternatively spliced murine PAPSS2 supported by the ESTs listed in **Table 2** displayed import activity. In agreement with the swapping construct described above, this signal was less potent in nuclear import than its PAPSS1 counterpart.

The N-terminal 33 amino acids are disordered in the crystal structure of the PAPSS1 full length protein [10]. In a recent structure of the isolated APS kinase domain of the same protein, nine additional amino acids are visible [30], still indicating the flexibility of the remaining 24 N-terminal amino acids. This special structural feature makes it unlikely that the N-terminal KKxK motif belongs to a classical bipartite NLS [31] or even to the newly described extended NLS [32], [33], though the elongated PAPSS1-NLS was slightly more active in microinjection experiments than its shorter counterpart.

We conclude that the N-terminal KKxK motifs in both PAPS synthases are functional nuclear localisation sequences. The dimeric context of PAPS synthases may increase the efficacy of this unusually short motif. Spacing and/or the exact nature of the surrounding amino acids may tune the N-terminus of PAPSS1 to a more effective nuclear localisation signal than the other, shorter sequence of PAPSS2.

### Conclusion

In the present study, we have characterised the localisation signals that govern cellular distribution of PAPS synthases, the key enzymes of biological sulphation reactions. Taking the molecular weight of 140 kDa of the high affinity PAPS synthase dimers into account [18], these enzymes seem to shuttle between cytoplasm and the cellular nucleus using active transport mechanisms. We have determined major localisation signals that influence cellular localisation patterns of human PAPS synthase isoforms. Moreover, we could derive relative strengths of the N-terminal KKxK NLS motifs in the two proteins. PAPS synthase 1 contains a stronger NLS than its PAPSS2 counterpart. Furthermore, we could identify a highly conserved Arg-Arg motif embedded within the α-helical APS kinase domain interface mediating export capacity. This long α-helical element around Arg111 and Arg112 in PAPSS1 is nearly identical in the amino acid sequence to the respective sequence in PAPSS2. Hence, this motif is expected to influence both enzyme isoforms to a similar extent. Transient binding of some yet unknown proteins or masking one of these counteracting signals by post-translational modifications may be an efficient way to adaptively relocate both PAPS synthases according to varying cellular needs. Our analysis of signals that regulate nucleocytoplasmic shuttling of human PAPS synthases suggests that both PAPSS isoforms need to be considered when addressing regulatory functions of these PAPS producing enzymes within the nucleus or the sulphation-dependent step in retroviral infection.

## Materials and Methods

### Plasmids and mutants

Coding sequences for human PAPS synthase 1 [RefSeq: NM_005443] and PAPSS2B [NM_001015880] have been described previously [10], [18]. They were inserted via NheI/BamHI into living colour vectors pEGFP-N1 and pDsRed_mono_-N1 [5]. Please refer to **Information S1** for all primers used in this study. The enhancer region of the CMV promoter in all pEGFP-PAPSS2B constructs contained a 269 nucleotides-comprising deletion that may be represented as partial cleavage with AatII and re-ligation and did not alter cellular localisation. All mutants were prepared by site-directed mutagenesis with DpnI digestion of the parental DNA (QuikChange kit, Stratagene, Agilent Technologies, Waldbronn, Germany). A swapping construct with the PAPSS1 N-terminus and the PAPSS2 body was generated by first introducing an AclI site (AA∧CGTT) via silent mutations of the Asn (aat→aaC) and Val codons (gtc→gtT) in both PAPS synthase-EGFP fusion vectors (codons 27/28 and 17/18 in PAPSS1 and -S2, respectively) followed by AclI/BamHI cloning of the PAPSS2 body into the PAPSS1-EGFP fusion vector. Potential localisation signals and domains were cloned into the bacterial expression vector pGEX-GFP in between GST and GFP by annealing of overlapping oligonucleotides comprising sticky BamHI and NheI restriction sites as described [22]. All resulting expression plasmids (**Information S1**) were verified by standard DNA sequencing.

### Cell culture, transfection, microinjection, and fluorescence imaging of cells

The human epithelial carcinoma cell line HeLa (ATCC-No. CCL-2), the human embryonic kidney cell line HEK293 cells (ATCC CRL-1573), Chinese hamster ovary CHO cells (ATCC CCL-61) as well as the monkey kidney cell line Vero (ATCC-No. CRL-1587) were maintained as recommended by the American Tissue Culture Collection (ATCC). HeLa cells grown on cover slips as described [34] were transiently transfected with FUGENE HD (Roche, Mannheim, Germany) according to the manufacturer's instructions using plasmid DNA purified with the NucleoBond Mini kit (Macherey-Nagel, Düren, Germany). Nuclear DNA was co-stained with Hoechst 33258. Leptomycin B (Alexis Biochemicals/Enzo Life Science, Lörrach, Germany) was used at a concentration of 10 nM for the indicated number of hours. Cover slips were fixed in 4% para-formaldehyde, mounted with Mowiol 4–88 on microscope slides and sealed using nail polish.

Fluorescent protein fusions of PAPS synthases were visually classified into five categories. Therefore, blinded samples were examined in each case. For all samples at least 200 cells were scored, otherwise counted cell numbers (n) are indicated. Standard deviations were between 6 and 8% when averaged over the three largest fractions. These errors were derived from evaluating same samples by the same individual on different days as well as same samples counted by different persons.

In a complementary, unbiased computation-based approach, quantitative localisation of EGFP fusion proteins was assessed after binarisation. Therefore, original images acquired with a 10× objective were binarised within the cell∧P software (Olympus, Münster, Germany) as previously described [34]. A total of six parameters was extracted from these images: area of Hoechst fluorescence, EGFP fluorescence and co-localisation, co-localisation relative to Hoechst and EGFP areas as well as the ratio of EGFP and Hoechst areas that reflects transformation efficiency. The area of co-localisation relative to the EGFP fluorescence was found to be a robust parameter for nuclear localisation of our PAPS synthase-EGFP fusion proteins. Fluorescence intensity of single cells was determined using ImageJ 1.45 s.

Purification and microinjection of recombinant GST-GFP transport substrates in Vero cells were performed as described in detail [22]. Observation, quantitation, image analysis and presentation were performed as described [35].

### Analytical gel filtration of PAPSS1 mutants

PAPS synthase 1 wild type and mutant proteins were expressed in *E. coli* expression strains as fusions to GST-His6-PreScission as previously described [18]. Site-directed mutagenesis was repeated here with the same oligonucleotides used for mutagenesis in the EGFP context. Proteins were purified on GSH sepharose. After digestion with PreScission protease, pure PAPSS protein was obtained on a Superdex 200 prep grade 26/60 (GE Healthcare, Freiburg, Germany). Analytical gel filtration was then performed on a Superdex 200 10/300 GL gel filtration column (GE Healthcare, Freiburg, Germany) equilibrated in 20 mM Tris (pH 7.3), 180 mM NaCl, 0.1 mM EDTA and 1 mM DTT that was calibrated using thyroglobulin, ferritin, catalase, aldolase, albumin and lysozyme.

### Bioinformatics

Structural investigation of existing PDB files was performed using YASARA [36]. Secondary structure predictions were done using NPS [21]. Surface exposure of particular amino acids was analysed using the DSSP tool [37] as described [18]. Sequences were aligned using ClustalW [38]. The mouse EST collection at GenBank was searched for using BLAST [39]. Genomic DNA sequence for the murine PAPSS2 locus ENSMUSG00000024899 was retrieved from Ensembl [40] and searched for using the bl2seq option of BLAST [39].

## Supporting Information

Information S1 **Cellular localisation of PAPS synthase proteins.** Localisation of different PAPS synthase fusion proteins in several cell types. The influence of the nature of the fusion protein (expressed in HeLa cells) as well as the cell line used was assayed for PAPSS1 and PAPSS2 fusion proteins. At least 200 cells were scored according to the schematic presented in Figure 1. **PAPS synthases contain a leucine-rich motif that acts as weak NES.** **A**, alignment of residues Leu254–Leu270 of PAPSS1 (P1) with the respective Leu244–Leu260 sequence of PAPSS2 (P2). The short and long peptidic motifs tested for their nuclear export activity are indicated. Residues of a putative leucine-rich NES are written in bold. NPS secondary structure consensus prediction is shown below the alignment; “.”, coil; h/H, weak/prominent helical propensity; “?”, no prediction. **B**, mapping of this putative leucine-rich export signal on one part of the ATP sulphurylase domain of the PAPSS1 crystal structure 1X6V. The short sequence is surface-exposed and shown in cyan, the extension in blue. **C**, recombinant GST-PAPSS1/2-NES-GFP proteins containing the respective leucine-rich signal sequences showed weak export activity upon microinjection into the nuclei of Vero cells at the indicated time points (left panels). In contrast, the GST-GFP substrate remained nuclear under the same experimental conditions (right panel). Approximately 50 cells were injected, and representative images from live-cell fluorescence microscopy are shown. **D**, due to the slow export kinetics of this motif it was not possible to monitor completion of export within one round of the cell cycle, therefore monitoring included mitotic nuclear envelope breakdown and reassembly. **Expression levels of PAPSS2-EGFP wild-type and mutant proteins.** Equal amounts of HeLa cells were transfected with wild-type PAPSS2-EGFP expression plasmid as well as those three mutants that showed the largest shifts in cellular localisation: KK6,8AA, RR101,102AA and LL252,254AA and expression levels were assessed by western blotting using an anti-GFP antibody. Equal loading was shown using an anti-tubulin antibody. All four fusion proteins showed similar expression levels at comparable rates of transfection. Additionally, detailed information on the plasmids and oligonucleotides used in this study can be found within Information S1.
(PDF)
