# Supplementary material for: Human PAPS Synthase Isoforms Are Dynamically Regulated Enzymes with Access to Nucleus and Cytoplasm
Source: PLoS One. 2012 Jan 5;7(1):e29559. doi: 10.1371/journal.pone.0029559 (PMC3252339; doi:10.1371/journal.pone.0029559)
Supplement: Information S1 — Cellular localisation of PAPS synthase proteins. Localisation of different PAPS synthase fusion proteins in several cell types. The influence of the nature of the fusion protein (expressed in HeLa cells) as well as the cell line used was assayed for PAPSS1 and PAPSS2 fusion proteins. At least 200 cells were scored according to the schematic presented in Figure 1. PAPS synthases contain a leucine-rich motif that acts as weak NES. A, alignment of residues Leu254–Leu270 of PAPSS1 (P1) with the respective Leu244–Leu260 sequence of PAPSS2 (P2). The short and long peptidic motifs tested for their nuclear export activity are indicated. Residues of a putative leucine-rich NES are written in bold. NPS secondary structure consensus prediction is shown below the alignment; “.”, coil; h/H, weak/prominent helical propensity; “?”, no prediction. B, mapping of this putative leucine-rich export signal on one part of the ATP sulphurylase domain of the PAPSS1 crystal structure 1X6V. The short sequence is surface-exposed and shown in cyan, the extension in blue. C, recombinant GST-PAPSS1/2-NES-GFP proteins containing the respective leucine-rich signal sequences showed weak export activity upon microinjection into the nuclei of Vero cells at the indicated time points (left panels). In contrast, the GST-GFP substrate remained nuclear under the same experimental conditions (right panel). Approximately 50 cells were injected, and representative images from live-cell fluorescence microscopy are shown. D, due to the slow export kinetics of this motif it was not possible to monitor completion of export within one round of the cell cycle, therefore monitoring included mitotic nuclear envelope breakdown and reassembly. Expression levels of PAPSS2-EGFP wild-type and mutant proteins. Equal amounts of HeLa cells were transfected with wild-type PAPSS2-EGFP expression plasmid as well as those three mutants that showed the largest shifts in cellular localisation: KK6,8AA, RR101,102AA and LL252,254AA [file pone.0029559.s001.pdf]

Human PAPS synthase isoforms are dynamically regulated enzymes with access to nucleus and cytoplasm

Running title: Nucleocytoplasmic shuttling of PAPS synthases

Elisabeth Schröder[&], Lena Gebel[&], Andrey A. Eremeev, Jessica Morgner, Daniel Grum, Shirley K. Knauer, Peter Bayer, and Jonathan W. Mueller

[&]Equally contributing authors.

Supporting Information

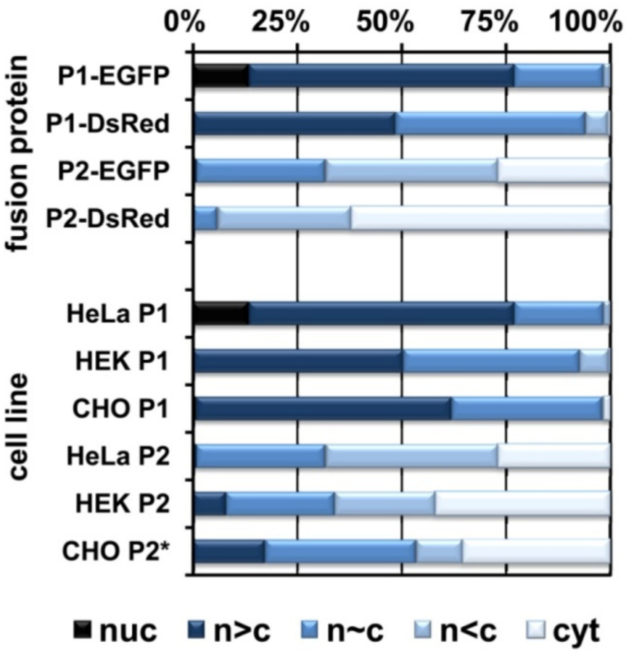

Supplementary figure 1: Cellular localisation of PAPS synthase proteins.
Localisation of different PAPS synthase fusion proteins in several cell types. The influence of the nature of the fusion protein (expressed in HeLa cells) as well as the cell line used was assayed for PAPSS1 and PAPSS2 fusion proteins. At least 200 cells were scored according to the schematic presented in Figure 1.
*Localisation of PAPSS2 in CHO cells was scored using a PAPSS2-DsRed fusion protein.

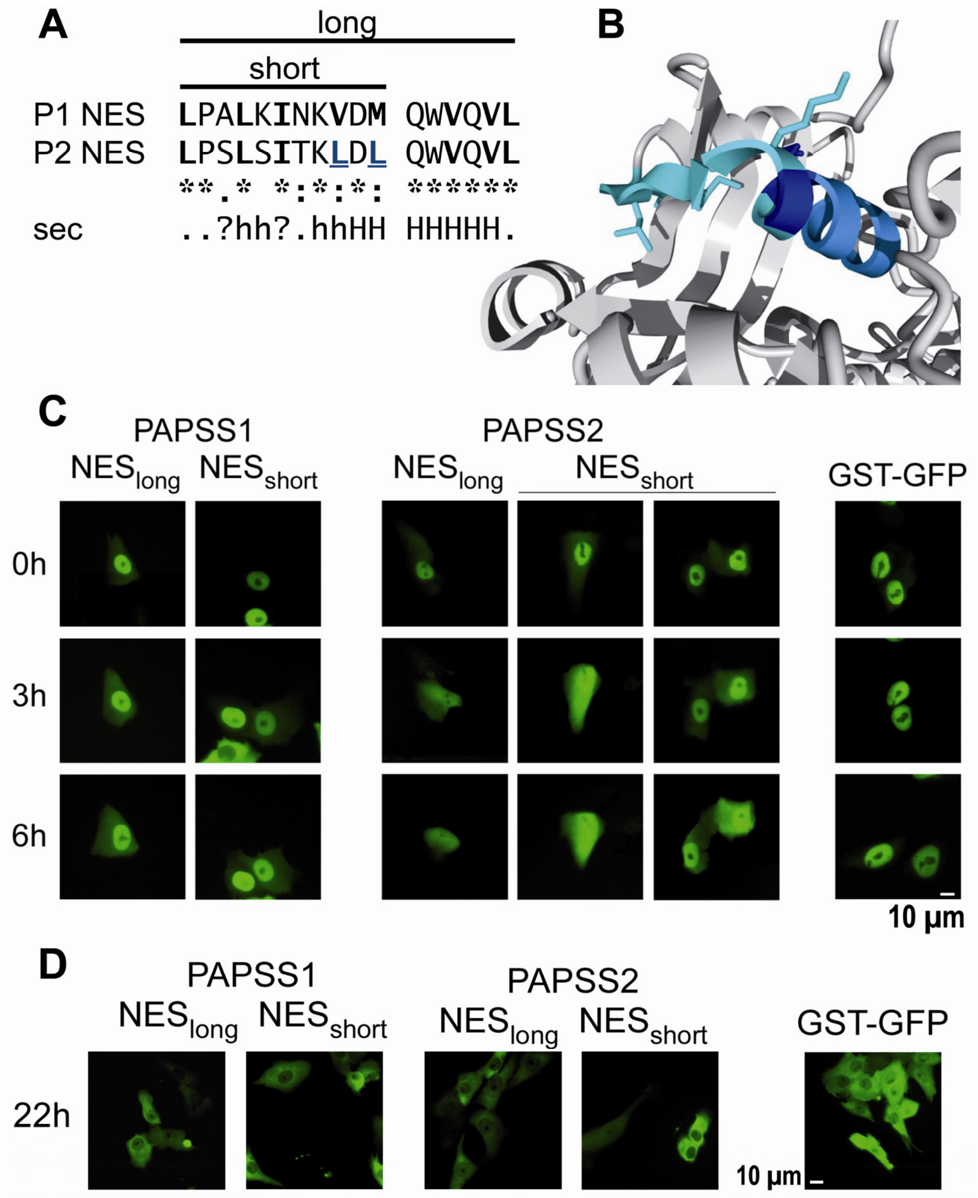

Supplementary figure 2: PAPS synthases contain a leucine-rich motif that acts as weak NES. **A**, alignment of residues Leu254-Leu270 of PAPSS1 (P1) with the respective Leu244-Leu260 sequence of PAPSS2 (P2). The short and long peptidic motifs tested for their nuclear export activity are indicated. Residues of a putative leucine-rich NES are written in bold. NPS secondary structure consensus prediction is shown below the alignment; ".", coil; h/H, weak/prominent helical propensity; "?", no prediction. **B**, mapping of this putative leucine-rich export signal on one part of the ATP sulphurylase domain of the PAPSS1 crystal structure 1X6V. The short sequence is surface-exposed and shown in cyan, the extension in blue. **C**, recombinant GST-PAPSS1/2-NES-GFP proteins containing the respective leucine-rich signal sequences showed weak export activity upon microinjection into the nuclei of Vero cells at the indicated time points (left panels). In contrast, the GST-GFP substrate remained nuclear under the same experimental conditions (right panel). Approximately 50 cells were injected, and representative images from live-cell fluorescence microscopy are shown. **D**, due to the slow export kinetics of this motif it was not possible to monitor completion of export within one round of the cell cycle, therefore monitoring included mitotic nuclear envelope breakdown and reassembly.

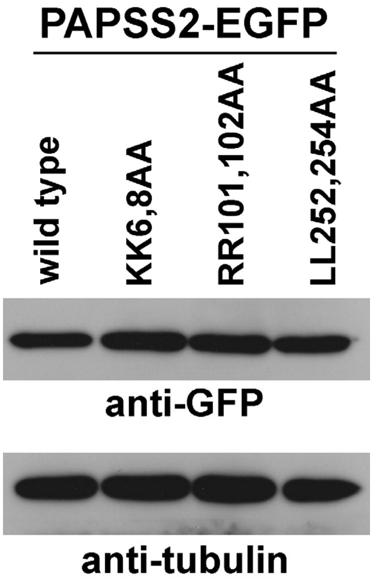

Supplementary figure 3: Expression levels of PAPSS2-EGFP wild-type and mutant proteins
Equal amounts of HeLa cells were transfected with wild-type PAPSS2-EGFP expression plasmid as well as those three mutants that showed the largest shifts in cellular localisation: KK6,8AA, RR101,102AA and LL252,254AA and expression levels were assessed by western blotting using an anti-GFP antibody. Equal loading was shown using an anti-tubulin antibody. All four fusion proteins showed similar expression levels at comparable rates of transfection.

Supplementary table 1: Plasmids used in this study

Name	Encoded construct (including tags)	Vector	Restriction sites/primer	Comments
wild-type constructs				
pBC828	PAPSS1-EGFP	pEGFP-N1	NheI/BamHI	
pBC850	PAPSS2B-EGFP	pEGFP-N1	NheI/BamHI	
	GST-His_6-PreScission-PAPSS1	pET-41-PreScission	ApaI/EcoRI	Grum et al., 2010
	PAPSS1-$DsRed_{mono}$			
	PAPSS2B- $DsRed_{mono}$			
PAPSS1 point mutants				
pBC889	KK9,10AA	pBC828	827/828	NLS
pBC859	RR111,112AA	pBC828	829/830	putative NLS
pBC885	KK175,176AA	pBC828	831/832	putative NLS within PAPS loop
pBC886	RR333,334AA	pBC828	833/834	putative NLS
pBC860	R339Q	pBC828	835/836	different from mouse PAPS synthase (Q)
pBC887	R442A	pBC828	837/838	putative NLS
pBC881	RK592,593AA	pBC828	843/844	putative NLS
pBC882	L119K	pBC828	720/721	interface mutant
pBC883	RR111,112 AA		829/830	for bacterial expression
	L119K			for bacterial expression
PAPSS2 point mutants				
pBC911	KK6,8AA	pBC850	849/850	NLS
pBC909	RR101,102AA	pBC850	851/852	putative NLS
pBC890	KR165,166AA	pBC850	853/854	putative NLS in the PAPS loop
pBC891	II221,224AA	pBC850	855/856	putative NES close to domain linker
pBC910	LL252,254AA	pBC850	859/860	putative NES different from P1
pBC897	RR328,329AA	pBC850	861/862	putative NLS

pBC892	R334Q	pBC850	863/864	different from mouse P2 (Q)
pBC893	L396T	pBC850	865/866	putative NES different from P1
pBC894	RR431,432AA	pBC850	867/868	putative NLS different from P1
pBC895	R437Ala	pBC850	869/870	putative NLS
pBC896	KK564,565AA	pBC850	871/872	putative NLS
other constructs				
				swapping
PAPSS1-NES$_{long}$	LPALKINKVDMQWVQVL	pGEX3-GFP	P1NESl5/P1NESl3	putative NES, for bacterial expression
PAPSS1-NES$_{short}$	LPALKINKVDM	pGEX3-GFP	P1NESs5/P1NESs3	putative NES, for bacterial expression
PAPSS2-NES$_{long}$	LPSLSITKLDLQWVQVL	pGEX3-GFP	P2NESl5/P2NESl3	putative NES, for bacterial expression
PAPSS2-NES$_{short}$	LPSLSITKLDL	pGEX3-GFP	P2NESs5/P2NESs3	putative NES, for bacterial expression
PAPSS1-Helix	SPEDREENVRRIAEVAKLFADAG	pGEX3-GFP	P1Hx5/P1Hx3	putative NES, for bacterial expression
PAPSS1-Helix$_{Mut}$	SPEDREENVAAIAEVAKLFADAG	pGEX3-GFP	P1HxM5/P1HxM3	putative NES, for bacterial expression
PAPSS1-NLS$_{long}$	MEIPGSLCKKVKLSNNAQNWGMQ RATNVTYQAHHVSRNKR	pGEX3-GFP	P1NLSl5/P1NLSl3	putative NLS, for bacterial expression
PAPSS1-NLS$_{short}$	MEIPGSLCKKVKLS	pGEX3-GFP	P1NLSs5/P1NLSs3	putative NLS, for bacterial expression
mPAPSS2-NLS	MSANFKMNHKR	pGEX3-GFP	P2mNLS5/P2mNLS3	putative NLS, for bacterial expression
mPAPSS2-NLS*	MSGVKKQKT	pGEX3-GFP	P2m*NLS5/P2m*NLS3	putative NLS, for bacterial expression
RevNES	LQLPPLERLTL	pGEX3-GFP	5'RevNES/3'RevNES	control NES, for bacterial expression
SV40NLS	PPKKKRKVEDP	pGEX3-GFP	5'SV40NLS/3'SV40NLS	control NLS, for bacterial expression

Supplementary table 2: Oligonucleotides used in this study

BC No.	Sequence	Content
541F	CAC ACA GCT AGC CAC CAT GGA GAT CCC CGG GAG CCT GTG C	forward PCR primer for human PAPSS1 with NheI, Kozak and ATG codon
542R	C ATG ATG GGC TTG GTA GGT AAC GTT GGT TGC TCT CTG CAT TCC C	mutagenesis primer for introduction of internal AclI site in PAPSS1
587F	G GGA ATG CAG AGA GCA ACC AAC GTT ACC TAC CAA GCC CAT CAT G	
543R	CAC ACA GGA TCC GCT TTC TCC AAG GAT TTG TAG TAT TC	reverse PCR primer for human PAPSS1 with BamHI site WITHOUT STOP codon
544R	CAC ACA GGA TCC TTA AGC TTT CTC CAA GGA TTT GTA GTA TTC	reverse PCR primer for human PAPSS1 with BamHI site AND STOP codon
545F	CAC ACA GCT AGC CACC ATG TCG GGG ATC AAG AAG CAA AAG	forward PCR primer for human PAPSS2 with NheI, Kozak and ATG codon
546R	C ATG GTG GGC CTG ATA GAC AAC GTT GGT GGA TTT CTG CTG GTT C	mutagenesis primer for introduction of internal AclI site in PAPSS2
588F	G AAC CAG CAG AAA TCC ACC AAC GTT GTC TAT CAG GCC CAC CAT G	
547R	CACACA GG ATC CCC GTT CTT CTC CAG GGA CCT GTA ATA ATC	reverse PCR primer for human PAPSS2 with BamHI site, additional Gly but no STOP codon
PAPSS1 point mutations for the NLS/NES signal screen		
720F	TCG CAG AAG TTG CTA AAA AGT TTG CAG ATG CTG GCT TAG	QC primer PAPSS1_L119K
721R	AAG CCA GCA TCT GCA AAC TTT TTA GCA ACT TCT GCG ATG	
722F	GTT TGC AGA TGC TGG CAA AGT GTG CAT CAC AAG TTT C	QC primer PAPSS1_L125K
723R	GAA ACT TGT GAT GCA CAC TTT GCC AGC ATC TGC AAA C	
724F	GAG CAC AGG AAA GAG TGG CGC TGT GCC AGA C	QC primer PAPSS1_E350W
725R	GTC TGG CAC AGC GCC ACT CTT TCC TGT GCT C	
827F	CCC GGG AGC CTG TGC GCC GCA GTC AAA CTG AGC AAT AAC GCG	QC primer PAPSS1_KK9,10AA
828R	CGC GTT ATT GCT CAG TTT GAC TGC GGC GCA CAG GCT CCC GGG	
829F	GAC AGA GAA GAG AAT GTT GCA GCC ATC GCA GAA GTT GCT AAA CTG	QC primer PAPSS1_RR111,112AA
830R	T AGC AAC TTC TGC GAT GGC TGC AAC ATT CTC TTC TCT GTC TTC AGG	
831F	GAT GTC AAA GGA CTC TAC GCA GCA GCC CGG GCA GGA GAA ATT AAA G	QC primer PAPSS1_KK175,176AA
832R	C TCC TGC CCG GGC TGC TGC GTA GAG TCC TTT GAC ATC CCT C	
833F	GCT CTG ATG TAT GAG GGC GCC GCT GTG GCC ATT CTT CGC AAT CC	QC primer PAPSS1_RR333,334AA
834R	GCG AAG AAT GGC CAC AGC GGC GCC CTC ATA CAT CAG AGC AAA TG	

835F	CGC CGT GTG GCC ATT CTT CAG AAT CCA GAG TTT TTT GAG CAC AGG	QC primer PAPSS1_R339Q
836R	CCT GTG CTC AAA AAA CTC TGG ATT CTG AAG AAT GGC CAC ACG GCG	
837F	CC CAT AAG CAA CTT CTA GAG GCC GGC TAC CGG CGC CCT G	QC primer PAPSS1_R442A
838R	C AGG GCG CCG GTA GCC GGC CTC TAG AAG TTG CTT ATG GGT ATC	
839F	CAA CTT CTA GAG AGG GGC TAC GCC GCC CCT GTC CTC CTC CTC C	QC primer PAPSS1_RR445,446AA
840R	G GAG GAG GAG GAC AGG GGC GGC GTA GCC CCT CTC TAG AAG TTG	
841F	GTT GCA GCT TAC AAC AAG GCA GCC GCC CGT ATG GAC TAC TAT GAC TCT G	QC primer PAPSS1_KKK568,569,570AAA
842R	GTC ATA GTA GTC CAT ACG GGC GGC TGC CTT GTT GTA AGC TGC AAC TCG	
843F	TT ATT TCA GGA ACA CGA ATG GCC GCA CTT GCT CGA GAA GGC CAG	QC primer PAPSS1_RK592,593AA
844R	GGC CTT CTC GAG CAA GTG CGG CCA TTC GTG TTC CTG AAA TAA ATT CAA AG	
845F	G CAG AGA GCA ACC AAT GTC GTC TAC CAA GCC CAT CAT GTC AG	QC primer PAPSS1_T29V
846R	GAC ATG ATG GGC TTG GTA GAC GAC ATT GGT TGC TCT CTG CAT TC	
847F	CG GGA AAG ACT ACT GTG AGC TTT GCC TTG GAG GAG TAC CTG	QC primer PAPSS1_M70F
848R	CAG GTA CTC CTC CAA GGC AAA GCT CAC AGT AGT CTT TCC CG	
PAPSS2 point mutations for the NLS/NES signal screen		
849F	C ATG TCG GGG ATC AAG GCC CAA GCC ACG GAG AAC CAG CAG AAA TC	QC primer PAPSS2_KK6,8AA in the pEGFP-N1 vector (with part of the Kozak sequence)
850R	CTG CTG GTT CTC CGT GGC TTG GGC CTT GAT CCC CGA CAT GTGG	
851F	G GAC AGA GAG GAA AAT ATC GCC GCC ATT GCT GAG GTG GCT AAG CTG	QC primer PAPSS2_RR101,102AA
852R	CTT AGC CAC CTC AGC AAT GGC GGC GAT ATT TTC CTC TCT GTC CCC	
853F	GAC GTA AAA GGC CTC TAT GCA GCC GCC AGA GCT GGG GAG ATT AAA G	QC primer PAPSS2_KR165,166AA
854R	CTC CCC AGC TCT GGC GGC TGC ATA GAG GCC TTT TAC GTC TCT GC	
855F	CAT TGT ACC CTA TAC TAT AGC CAA AGA TGC CCA CGA ACT CTT TGT GCC G	QC primer PAPSS2_II221,224AA
856R	GCA CAA AGA GTT CGT GGG CAT CTT TGG CTA TAG TAT AGG GTA CAA TGT TCT G	
857F	GCT GAA ACT CTC CCT TCA GCA TCA GCT ACT AAG CTG GAT CTC CAG TGG	QC primer PAPSS2_LI247,249AA
858R	CTG GAG ATC CAG CTT AGT AGC TGA TGC TGA AGG GAG AGT TTC AGC CTC	
859F	CTT CAT TAT CAA TTA CTA AGG CCG ATG CCC AGT GGG TCC AGG TTT TGA G	QC primer PAPSS2_LL252,254AA
860R	AAC CTG GAC CCA CTG GGC ATC GGC CTT AGT AAT TGA TAA TGA AGG GAG AG	
861F	GTC CTG GCA CAT GGT GGA GCC GCC GTA GCT ATC TTA CGA GAC GCT G	QC primer PAPSS2_RR328,329AA
862R	C AGC GTC TCG TAA GAT AGC TAC GGC GGC TCC ACC ATG TGC CAG GAC	
863F	GA CGG AGG GTA GCT ATC TTA CAA GAC GCT GAA TTC TAT GAA CAC AG	QC primer PAPSS2_R334Q
864R	CT GTG TTC ATA GAA TTC AGC GTC TTG TAA GAT AGC TAC CCT CCG TC	

865F	TAC CGT CTG ACA CCT ACC GAG CTC AAA CAG AAA TGT AAA GAA ATG	QC primer PAPSS2_L396T
866R	C TTT ACA TTT CTG TTT GAG CTC GGT AGG TGT CAG ACG GTA TTG GTC	
867F	CC CTG TTG ATG CAG GAC ACT GCC GCC AGG CTC CTA GAG AGG GG	QC primer PAPSS2_RR431,432AA
868R	CC CCT CTC TAG GAG CCT GGC GGC AGT GTC CTG CAT CAA CAG GG	
869F	GC CGC AGG CTC CTA GAG GCC GGC TAC AAG CAC CCG GTC	QC primer PAPSS2_R437A
870R	GAC CGG GTG CTT GTA GCC GGC CTC TAG GAG CCT GCG GC	
871F	CT GCC TAC AAC AAA GCC GCA GCA GCC ATG GAC TTC TAT GAT CCA G	QC primer PAPSS2_KK564,565AA
872R	G ATC ATA GAA GTC CAT GGC TGC TGC GGC TTT GTT GTA GGC AGC CAC	
873F	C TTC ATC TCA GGA ACT CGA ATG GCC GCC CTC GCC CGG GAA GGA G	QC primer PAPSS2_RK587,588AA
874R	C TCC TTC CCG GGC GAG GGC GGC CAT TCG AGT TCC TGA GAT GAA G	
875F	C CAG CAG AAA TCC ACC AAT GTA AAC TAT CAG GCC CAC CAT GTG AG	QC primer PAPSS2_V19N
876R	CT CAC ATG GTG GGC CTG ATA GTT TAC ATT GGT GGA TTT CTG CTG G	
877F	GGT GCT GGA AAA ACA ACG ATA AGT ATG GCC CTG GAG GAG TAC CTT G	QC primer PAPSS2_F60M
878R	C AAG GTA CTC CTC CAG GGC CAT ACT TAT CGT TGT TTT TCC AGC ACC	
913F	C CAG CAG AAA TCC ACC AAT GTA ACC TAT CAG GCC CAC CAT GTG AG	QC primer PAPSS2_V19T
914R	CT CAC ATG GTG GGC CTG ATA GGT TAC ATT GGT GGA TTT CTG CTG G	
Oligonucleotides for GST-signal-peptide-GFP fusions in pGEX vectors		
P2NESs5	GAT CCA CCT CCC TTC ATT ATC AAT TAC TAA GCT GGA TCT CG	for insertion of PAPSS2-NES$_{short}$ into pGEX-GST-GFP via BamHI/NheI
P2NESs3	CTA GCG AGA TCC AGC TTA GTA ATT GAT AAT GAA GGG AGG TG	
P2NESl5	GAT CCA CCT CCC TTC ATT ATC AAT TAC TAA GCT GGA TCT CCA GTG GGT CCA GGT TTT GG	for insertion of PAPSS2-NES$_{long}$ into pGEX-GST-GFP via BamHI/NheI
P2NESl3	CTA GCC AAA ACC TGG ACC CAC TGG AGA TCC AGC TTA GTA ATT GAT AAT GAA GGG AGG TG	
P1NESs5	GAT CCA CTT ACC AGC ACT GAA AAT TAA TAA AGT GGA TAT GG	for insertion of PAPSS1-NES$_{short}$ into pGEX-GST-GFP via BamHI/NheI
P1NESs3	CTA GCC ATA TCC ACT TTA TTA ATT TTC AGT GCT GGT AAG TG	
P1NESl5	GAT CCA CTT ACC AGC ACT GAA AAT TAA TAA AGT GGA TAT GCA GTG GGT GCA GGT TTT GG	for insertion of PAPSS1-NES$_{long}$ into pGEX-GST-GFP via BamHI/NheI
P1NESl3	CTA GCC AAA ACC TGC ACC CAC TGC ATA TCC ACT TTA TTA ATT TTC AGT GCT GGT AAG TG	
P1Hx5	GAT CCA CAG TCC TGA AGA CAG AGA AGA GAA TGT TCG ACG CAT CGC AGA AGT TGC TAA ACT GTT TGC AGA TGC TGG CG	for insertion of PAPSS1-Helix into pGEX-GST-GFP via BamHI/NheI
P1Hx3	CTA GCG CCA GCA TCT GCA AAC AGT TTA GCA ACT TCT GCG ATG CGT CGA ACA TTC TCT TCT CTG TCT TCA GGA CTG TG	

P1HxM5	GAT CCA CAG TCC TGA AGA CAG AGA AGA GAA TGT TGC CGC TAT CGC AGA AGT TGC TAA ACT GTT TGC AGA TGC TGG CG	for insertion of PAPSS1-Helix$_{Mut}$ into pGEX-GST-GFP via BamHI/NheI
P1HxM3	CTA GCG CCA GCA TCT GCA AAC AGT TTA GCA ACT TCT GCG ATA GCG GCA ACA TTC TCT TCT CTG TCT TCA GGA CTG TG	
P1NLSl5	TTT GGA TCC ACA TGG AGA TCC CCG GGA GC	for insertion of PAPSS1- NLS$_{long}$ into pGEX-GST-GFP via BamHI/NheI following PCR amplification
P1NLSl3	AAA GCT AGC TCT CTT GTT CCT GCT GAC	
P1NLSs5	GAT CCA CAT GGA GAT CCC CGG GAG CCT GTG CAA GAA AGT CAA ACT GAG CG	for insertion of PAPSS1- NLS$_{short}$ into pGEX-GST-GFP via BamHI/NheI
P1NLSs3	CTA GCG CTC AGT TTG ACT TTC TTG CAC AGG CTC CCG GGG ATC TCC ATG TG	
P2mNLS5	GAT CCA CAT GTC TGC AAA TTT CAA AAT GAA CCA TAA AAG AGA CG	for insertion of murine PAPSS2-NLS into pGEX-GST-GFP via BamHI/NheI
P2mNLS3	CTA GCG TCT CTT TTA TGG TTC ATT TTG AAA TTT GCA GAC ATG TG	
P2m*NLS5	GAT CCA CAT GTC GGG GGT CAA GAA GCA GAA GAC GGA CG	for insertion of murine isoform PAPSS2-NLS* into pGEX-GST-GFP via BamHI/NheI
P2m*NLS3	CTA GCG TCC GTC TTC TGC TTC TTG ACC CCC GAC ATG TG	
5'RevNES	GAT CGA CGG CGG CCT TCA GCT ACC ACC GCT TGA GAG ACT TAC TCT TGG AG	for insertion of HIV1 RevNES into pGEX-GST-GFP via BamHI/NheI
3’RevNES	CTA GCT CCA AGA GTA AGT CTC TCA AGC GGT GGT AGC TGA AGG CCG CCG TC	
5'SV40NLS	GAT CGA CGG TAC TCC ACC TAA GAA AAA AAG AAA GGT TGA AGA TCC TGG AG	for insertion of SV40 NLS into pGEX-GST-GFP via BamHI/NheI
3’SV40NLS	CTA GCT CCA GGA TCT TCA ACC TTT CTT TTT TTC TTA GGT GGA GTA CCG TC	
